# A Young Man With Tuberculosis in the Neck Soft Tissue: A Case Report

**DOI:** 10.7759/cureus.66330

**Published:** 2024-08-06

**Authors:** Xiuming Wang, Haomei Luan, Zheyuan Zhang, Huabin Zhang

**Affiliations:** 1 Department of Ultrasound, Beijing Tsinghua Changgung Hospital, School of Clinical Medicine, Tsinghua University, Beijing, CHN

**Keywords:** extrapulmonary tuberculosis, cutaneous tuberculosis, soft tissue tuberculosis, case report, tuberculosis

## Abstract

Tuberculosis (TB) is a preventable and curable disease. TB characteristically causes lung infections, giving rise to pulmonary TB. Many extra-pulmonary organs, including soft tissues, may also be affected, often resulting in non-specific clinical features that make disease diagnosis difficult. We present the case of a 28-year-old male who presented with a soft tissue mass in the left neck accompanied by local redness and tenderness for several months. Despite initially erroneous clinical judgment and imaging diagnosis, the progression of the patient's disease combined with a series of laboratory tests led to the diagnosis of soft tissue TB. After routine anti-TB treatment, the patient's condition gradually recovered. This case highlights that when faced with atypical soft tissue lesions, physicians should maintain a high level of TB suspicion to avoid delaying the treatment of the patient's disease and producing a poor prognosis.

## Introduction

Tuberculosis (TB) is a curable disease caused by *Mycobacterium tuberculosis*. The main transmission route for TB is through the air. TB patients can release droplets containing *Mycobacterium tuberculosis* into the air when they cough, sneeze, or speak loudly. If healthy people inhale these droplets, they can become infected [1]. In 2022, TB will be the second-leading cause of death from a single infectious source in the world. It is reported that 7.5 million people will be newly diagnosed with TB in 2022, which is the highest number since 1995 [1]. By 2022, global TB cases will be mainly concentrated in developing countries, with India (27%), Indonesia (10%), and China (7.1%) ranking first [1].

Pulmonary TB occurs when the lungs are infected with *Mycobacterium tuberculosis*. Many extra-pulmonary organs, including soft tissues, may also be affected, often resulting in non-specific clinical features that make disease diagnosis difficult [2]. Complex clinical manifestations, including the variable site of the disease, local soft tissue redness and pain, mild systemic symptoms (only a few cases have night sweats), and so on, often lead to delayed diagnosis and improper treatment. Imaging plays a key role in disease discovery, diagnosis confirmation, and disease response monitoring [3]. We present a rare case of neck soft tissue TB, which is gradually diagnosed as the disease progresses. After active anti-TB therapy, the patient achieved a good prognosis.

## Case presentation

The study was approved by the Institutional Ethics Board of Beijing Tsinghua Changgung Hospital. Informed consent was obtained from the patient for the publication of all images, clinical data, and other data included in this study.

Since February 12, 2023, a 28-year-old unmarried male has discovered subcutaneous redness and swelling in his left neck. There is tenderness in the affected area and discomfort during movement. The patient has no fever, slight night sweats, fatigue, or decreased appetite. Local symptoms did not improve after two courses of empirical antibiotics, and the patient's condition continued. Therefore, the patient was admitted to the infection department of our hospital on May 16, 2023. A physical examination by the outpatient doctor revealed a palpable mass on the left side of the patient's neck. The epidermis of the mass was red, swollen, hard, and irregular. The size of the mass was about 7 x 3 cm, and the local tenderness was positive. The patient had been in good health and had no history of TB. The patient had no other skin lesions or rashes on his body. Additionally, this was the first time that he had presented with such swelling. On December 20, 2022, the patient experienced a COVID-19 infection, which was classified as mild. The patient took Lianhua Qingwen capsules and ibuprofen for symptomatic treatment and recovered well. The patient is otherwise healthy, with no known chronic diseases, immune disorders, or such. He has had no contact with sick patients and no recent travels. There was also no contact with animals. His systemic review was unremarkable.

Laboratory examination

On May 16, 2023, the blood routine examination and erythrocyte sedimentation rate of the patients were normal, and TB-SPOT-γ-interferon (T-N) indicated positive (13.32) pg/ml. On May 23, 2023, the patient had a positive purified protein derivative (PPD) test with an induration diameter of about 10 mm. There was also an elevated erythrocyte sedimentation rate of 107 mm/h (normal range: 0-17 mm/h). The TB antibody test (interferon-gamma release test) was positive. On June 6, 2023, the skin on the left side of the neck was ruptured, and caseous pus flowed out. The TB DNA test of the pus was positive.

Imaging examinations

**Figure 1:**
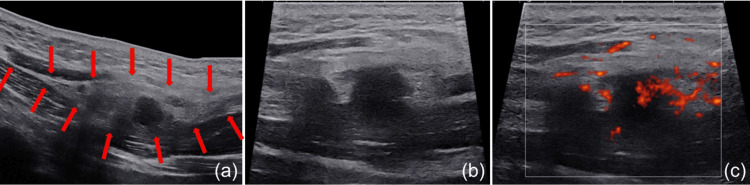
First ultrasound findings in our hospital on March 1, 2023 The superficial fascia of the left levator scapulae muscle was thickened, appearing as a fusiform mass with a scope of approximately 5.9 × 1.7 cm. A low echo area was visible at its center, with unclear boundaries, and the edges of the mass extended along the fascia. Ultrasound power Doppler suggested that the lesion had relatively rich blood flow signals. The ultrasound examination reported that the lesion was possibly nodular fasciitis. (a, panoramic ultrasound imaging of the lesion; b, extended field-of-view ultrasound imaging of the lesion, B-mode ultrasound; c, extended field-of-view ultrasound imaging of the lesion, power Doppler imaging)

**Figure 2:**
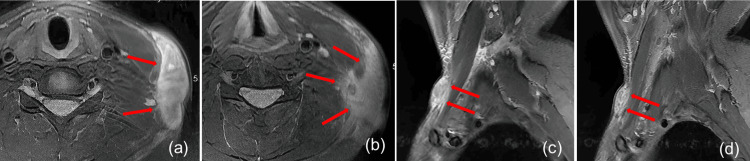
MRI findings on April 8, 2023 On the surface of the left neck, an irregular mass is visible, mainly involving the subcutaneous tissue but with an unclear boundary with the deep fascia, and some areas have invaded the deep fascia. In cross-section, the size of the mass is about 8.9 x 2.1 cm, with an up-down diameter of approximately 2.0 cm. It shows a high signal on T2WI. The MRI examination reported that the mass in the left neck may be nodular fasciitis. (a, b, cross-section; c, d, sagittal section) MRI: magnetic resonance imaging

**Figure 3:**
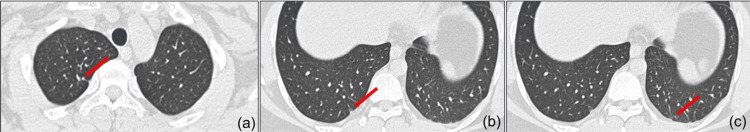
CT findings on May 19, 2023 There is a fibrotic sclerosis lesion in the upper lobe of the right lung, which is considered to be an old inflammatory lesion (a, red arrow). There are micro-nodules in both lungs, with the largest one being about 0.4 cm in diameter in the lower lobe of the right lung (b, red arrow). There is a small amount of inflammation in the lower lobe of the left lung (c, red arrow). CT: computed tomography

**Figure 4:**
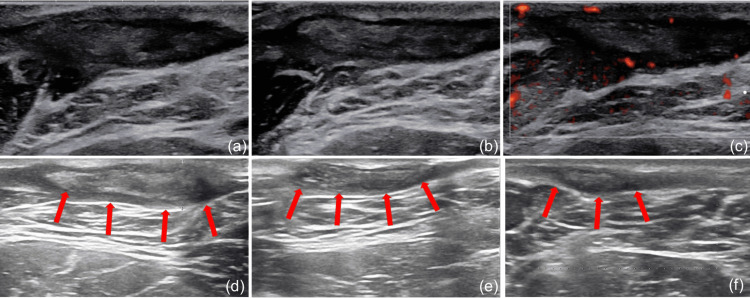
Ultrasonic follow-up after anti-TB treatment August 17, 2023 (a-c): The subcutaneous soft tissue of the left neck presents a patchy inhomogeneous hypoechoic area with a range of approximately 3.6 × 0.9 cm and increased blood flow signals. November 23, 2023 (d): An irregular strip-shaped hypoechoic area is visible under the skin of the left neck, with a thickness of approximately 0.7 cm, and no obvious blood flow signals are observed within it. March 28, 2024 (e, f): An irregular strip-shaped hypoechoic area is visible under the skin of the left neck, with a thickness of approximately 0.45 cm, and no obvious blood flow signals are observed within it. TB: tuberculosis

The ultrasound, CT, and MRI results of the patient during treatment in our hospital are shown in Figures 1-4.

Treatment

On May 23, 2023, the patient began anti-TB treatment with the anti-TB drugs isoniazid + rifampicin + moxifloxacin. On June 6, 2023, the patient was definitively diagnosed with TB infection and switched to the four-drug combination of isoniazid + rifampicin + moxifloxacin + pyrazinamide anti-TB therapy. From September 2023 to April 2024, repeated visits found that the skin rupture in the front of the neck gradually healed, the skin redness and swelling gradually improved, and the multiple nodules in the neck gradually shrank. On April 30, 2024, the patient developed abnormal liver function, and the anti-TB drugs were changed to pasiniazide + rifapentine, which have been used ever since. The patient has had regular liver function and blood-related laboratory tests since taking the medication.

## Discussion

The most common systemic symptoms of TB include fever, night sweats, fatigue, loss of appetite, and weight loss [4]. Extrapulmonary TB usually lacks typical clinical manifestations of a pauci-bacillary nature and is often overlooked by clinical doctors, leading to delayed diagnosis. Extrapulmonary TB is known as a great disease imitator because its clinical manifestations are highly variable, and patients often exhibit specific symptoms related to the affected organs [4,5]. The musculoskeletal system is usually infected in patients with extrapulmonary TB, accounting for 25% of cases [3].

Cutaneous and soft tissue TB accounts for 1% to 2% of extrapulmonary TB cases [6,7]. It most commonly manifests on the face, followed by the neck and trunk [8]. The present patient is a young male with no history of TB infection. A chest CT scan at admission does not confirm the presence of pulmonary TB, and he had a history of novel coronavirus infection prior to onset. Decreased immune function following COVID-19 infection provides an opportunity for *Mycobacterium tuberculosis* invasion and multiplication, likely the primary cause of TB granuloma development.

The early clinical symptoms of skin and soft tissue TB are usually atypical and easily confused with inflammatory lesions caused by bacterial or fungal infections [9]. Imaging also lacks specific features, and the low incidence rate of this disease contributes to a high early misdiagnosis rate [10]. In this case, it took up to two months from onset to diagnosis. Prior to admission to our hospital, the patient had visited multiple departments and was treated with routine antimicrobial dressings without improvement. Initial ultrasound and MRI scans misdiagnosed the lesion as nodular fasciitis.

The diagnosis of soft tissue TB is usually based on medical history, morphological features, laboratory tests, and histopathological features [9,11], but due to the similarity of these lesions to other skin diseases, the diagnosis may be complex, the diagnosis process may be long, and there may be significant delays. For patients presenting with unexplained subcutaneous soft tissue masses, apart from a thorough history and physical examination, TB-related indicators should be tested. Definitive diagnosis of cutaneous and soft tissue TB ultimately relies on skin biopsy and the isolation of *Mycobacterium tuberculosis* in culture. Typical TB lesions found on skin biopsy are epithelioid granulomas with central caseous necrosis that may or may not contain acid-fast bacilli (AFB). Other diagnostic tests include collecting sputum from AFB using Ziehl Nielsen staining, detecting *Mycobacterium tuberculosis* using sputum, blood, or tissue culture methods, the interferon-gamma release test, and the tuberculin skin test (TST, Mantoux, PPD) [12,13]. Compared with traditional smear and mycobacterial culture methods, polymerase chain reaction has the advantages of high specificity, sensitivity, speed, and simplicity [12], which is beneficial for the early diagnosis of extrapulmonary TB. The interferon-gamma release test has high sensitivity and specificity in diagnosing *Mycobacterium tuberculosis* [13], avoiding unnecessary trauma to patients and providing a valuable reference for early diagnosis of extrapulmonary TB. In this case, the patient's TB-SPOT-interferon-gamma (T-N) test was positive at 13.32 pg/ml, and the PPD test was also positive. As the disease progressed, the skin surface developed ulcerations with cheese-like necrotic secretions, and the TB antibody in the pus was positive with an ESR of 107. Gradually, the diagnosis of cutaneous and soft tissue TB was confirmed.

Anti-TB treatment is the main treatment method for extrapulmonary TB. Some experts believe that the treatment plan for extrapulmonary TB is similar to that for pulmonary TB [14]. However, there is no unified standard for the duration of treatment, which ranges from six months to one year. The treatment of skin and soft tissue TB follows the same guidelines as other organ TB. It includes a preliminary bactericidal treatment for two months using a combination of four drugs: rifampicin, isoniazid, pyrazinamide, and ethambutol (referred to as RIPE) or streptomycin. After this initial stage, there is a continuous phase that includes two drug regimens for four months (isoniazid and rifampicin daily or two to three times a week) [15-18]. Depending on the type of cutaneous and soft tissue TB and the immunologic status of the patient, therapy may need to be extended to nine months or even several years. In this case, the patient received anti-TB treatment with a combination of isoniazid, rifampin, moxifloxacin, and pyrazinamide. Surgical removal was considered risky due to the proximity of large vessels, such as the carotid artery, to the deep lesion. Given the good response to conventional anti-TB therapy, the patient underwent this treatment approach. The patient was satisfied with the results and agreed to be treated only with anti-TB drugs.

## Conclusions

Cutaneous and soft tissue TB is relatively rare, lacks characteristic clinical manifestations, and is difficult to diagnose early, often leading to misdiagnosis. Many patients undergo long-term routine antimicrobial and dressing changes without improvement before suspecting TB infection and undergoing relevant laboratory tests for a final diagnosis. Simultaneously, there is no standardized treatment protocol for this condition, and management often relies on doctors' individual treatment experiences. In this case, conventional anti-TB therapy with isoniazid, rifampin, moxifloxacin, and pyrazinamide achieved good outcomes and is worthy of promotion.
